# The American Medical Association

**Published:** 1872-04

**Authors:** 


### THE AMERICAN MEDICAL ASSOCIATION.

Since the last meeting of our Great National Medical Congress, in California, we have observed, in many of the Medical Journals of the country, a disposition to under-rate and under-value the dignity and high authority of that organization. In view of these murmurings and complainings, no more interesting study can engage the attention of the profession, for the vindication of the principles and the grand objects of the Association, than that of its past record and history. From the first, when—even before its organization—in the midst of great opposition and unjust reproaches, it announced its unrivalled Code of Medical Ethics, and its purpose to reform, to the utmost of its power, the ruinous inefficiency and manifold evils of the then prevailing methods of Medical teaching, both by preceptors and Colleges, to the present moment, it has been the mark of ridicule and unprofessional assault. Yet, from its origin to the present time, it has put upon record its devotedness to its original principles, at each recurring Annual Meeting. From the beginning, it has urged a higher standard of Medical Education, as the basis of professional integrity and dignity in this country. And it is due to truth and justice to say, that not only its origin but its past history, in the noble effort to elevate the standard of Medical Education, was the work, in a large measure, of the representatives of the first Medical Colleges of the land. The oldest Colleges of the Union have always united in giving tone and dignity to the American Medical Association; and while the efforts of their distinguished men, with those of other able members of the profession, not connected with our Medical Institutions, have failed in a perfect realization of their hopes; yet, the good accomplished by them, through the Association, has been of incalculable value to the profession. We have only to look at the rapid strides the profession were making under the selfish and ruinous guidance of rival Medical schools and of Medical educators—hopelessly drifting, without organization and without a Code of Morals to direct it, surely and rapidly toward the malestrom of professional degeneracy and ruin—and contrast what *must* have been, with what exists to-day, to measure the triumphs, and award that meed of praise due the American Medical Association. If these noble men, who, as representatives of our Medical Institutions, took the initial step in their grand effort to exalt the standard of Medical education, have failed to accomplish all they desired, the fault is not so much due to them or the organization they aided in bringing into existence, as to *outsides* (in heart) who, from the commencement opposed their efforts, and to the mass of the profession, who practically ignored the recommendations of the Association in preparing uneducated students for Collegiate honors.

From various quarters it is asserted that the American Medical Association has been given up to stormy debates, and that its members meet to quarrel to the neglect and sacrifice of the great interests of Science. While we regret to know that there may be some seeming truth in these charges, yet the fact does not denote degeneracy. On the contrary, these discussions may betray the existence of antagonistic forces in its membership, striving for mastery—the one on the line of principle, the other on that of expediency. To contend, to engage in controversy, is not disgraceful or unnecessary; and the champions of truth cannot conscientiously avoid the gage of battle when challenged by those who hold and proclaim error. We do not charge that any member or members of the Association have thrust erroneous doctrines upon that body. We desire, simply, to show that controversy, as such, is not harmful or hurtful to any cause; nay, that without controversy, no great cause has ever succeeded or can hope to succeed. The Christian is commanded to "*earnestly*"—yes "EARNESTLY" "contend for the faith once delivered to the Saints," and he would be a renegade in heart and practice, who, as a Christian, should fail to so "contend." It is not *contention* that becomes disreputable, and which stamps infamy upon men; but it is those contending in the interests of *wrong*, and by clothing the *wrong* with power sufficient to overthrow *principle*, thus crushing out the vital spark of truth, that degrades humanity and brings reproach upon any organization. Resistance to error—conscious advocacy of principle, is the key-note of Christian civilization, and is the foundation-stone of every Moral and Scientific organization. The Savior of man met His antagonists in controversy, and overcame them by the power of truth. His religion is built upon controversy, which the errors and evil combinations of men called forth in the Apostolic era; and the startling prophecy made to the little band of disciples before His crucifixion, that He "would not leave them peace, but a Sword," has been verified in every land since that day.

The charge, therefore, of quarrelsomness, made against the Association, if true, is shorn of its moral force, except when applied to those of that body, alone, who may seek to pervert its principles and destroy its integrity. That the charge is true, we are loth to believe. Not all discussions, yclept, "stormy," are necessarily the results of bad temper, nor are they indicative of a want of fealty to principle and truth. Truth is evolved by discussion—discussion directed in the interests of a righteous cause by righteous men.

Now, that discussions (some of which we have not approved), have latterly marked the history of the Association, we do not deny; but these discussions must form, until the Association is reconstructed, a part of its proceedings. The peculiar organization of the body has made it, in spite of a disposition on the part of the better portion of the membership to the contrary, a tribunal before which a host of professional grievances must annually be presented. It cannot be otherwise; and while it is right and proper that the highest Medical organization in the country should be made by common consent, the Supreme Medical Court of the profession—the finality of whose decisions should control—yet these difficulties should be placed, not before the body, but in the hands of a "Court of Arbitrators," composed of honest men from every State of the Union.

The originators of the Association contemplated a pure and elevated community of high-toned and christian gentlemen; and from this stand-point the organization was highly complimentary to the true professional men of the country—one uniting their interests and giving expression to the wants and feelings of the profession.

We do not despair, as some of the great National Medical organization. It is a professional necessity, in view of the rapid strides in the *regular* profession, to irregularities and charlatanism. Nothing but organization—effective, pure organization—based upon the eternal principles of truth and honor, can rescue the profession from ruin.

On the other hand, we regret to see that partyism more than adhesion to the grand and glorious truths embodied in Medical ethics, too often controls. Medical men too frequently, for interest, bury the professional gentleman in order to play the Medical demagogue. Such men rush into difficulties and take sides with motives more akin to the thorough-bred politician—destitute of principle—than a high-toned, pure-souled professional gentleman. Party strength, more than ethical truth and pure morality, govern and control them. The true man—whether physician or citizen, never "*takes sides*" until—like all the moral heroes of the past who have thrown a halo of glory along the pathway of truth—he is convinced, mind and heart, that he is right. Such a man is more to be admired than the proudest monarch.

Honest men never yield up principle to the demands of expediency or policy. They have an abiding faith in the power, and ultimate triumph of right, justice and truth. In the long-run, falsehood, though for the moment in the ascendent, must quail before the majesty of truth. For this reason, truth—the only foundation of honesty and good character—is the motive power of every holy purpose and every grand achievement. The conscientious man does not espouse it for *policy*, but because a nobler principle breathes its inspiration within him. A divinity that leads him, as a willing devotee, to the shrine of everlasting, immovable, unchangeable truth.

We therefore call upon the true, honest men of the profession, all over the land, to lend a helping hand to elevate the profession, by giving tone and dignity to the American Medical Association. Under the impulse of a holy energy, let them determine to make it the Standard of Right and Truth. If necessary, let some of their best, wisest, purest men of every State, be authorized by it to act as a Board of Arbitrators upon all questions involving a discussion of Medical Ethics, and individual action flowing therefrom. Let the requirements for membership be high—so high that a member will not scruple to sign himself "a member of the American Medical Association." In this way, we verily believe a line of demarkation may be authoritatively drawn between the honest, faithful, peaceful members of the profession, and the charlatans, the factionists and disorganizers; between the men who valiantly and nobly contend for the right, and those who seek to degrade the profession and overthrow its principles. Let the good men, thus, by a grand, moral, united effort proclaim the truth as the great, and only cementing power of the profession; and let them honor all who, fearlessly and zealously have upheld its standard in a noble contest against its enemies, amidst the frowns of a deluded populance, and the sneers and gibes of professional demagogues and charlatans.

